# Comparative Biomechanical Modeling of Metatherian and Placental Saber-Tooths: A Different Kind of Bite for an Extreme Pouched Predator

**DOI:** 10.1371/journal.pone.0066888

**Published:** 2013-06-26

**Authors:** Stephen Wroe, Uphar Chamoli, William C. H. Parr, Philip Clausen, Ryan Ridgely, Lawrence Witmer

**Affiliations:** 1 School of Biological, Earth and Environmental Sciences, University of New South Wales, Sydney, NSW, Australia; 2 School of Engineering, University of Newcastle, Callaghan, NSW, Australia; 3 St. George Clinical School, University of New South Wales, Sydney, NSW, Australia; 4 Department of Biomedical Sciences, Heritage College of Osteopathic Medicine, Ohio University, Athens, Ohio, United States of America; Monash University, Australia

## Abstract

Questions surrounding the dramatic morphology of saber-tooths, and the presumably deadly purpose to which it was put, have long excited scholarly and popular attention. Among saber-toothed species, the iconic North American placental, *Smilodon fatalis,* and the bizarre South American sparassodont, *Thylacosmilus atrox*, represent extreme forms commonly forwarded as examples of convergent evolution. For *S. fatalis*, some consensus has been reached on the question of killing behaviour, with most researchers accepting the canine-shear bite hypothesis, wherein both head-depressing and jaw closing musculatures played a role in delivery of the fatal bite. However, whether, or to what degree, *T. atrox* may have applied a similar approach remains an open question. Here we apply a three-dimensional computational approach to examine convergence in mechanical performance between the two species. We find that, in many respects, the placental *S. fatalis* (a true felid) was more similar to the metatherian *T. atrox* than to a conical-toothed cat. In modeling of both saber-tooths we found that jaw-adductor-driven bite forces were low, but that simulations invoking neck musculature revealed less cranio-mandibular stress than in a conical-toothed cat. However, our study also revealed differences between the two saber-tooths likely reflected in the *modus operandi* of the kill. Jaw-adductor-driven bite forces were extremely weak in *T. atrox*, and its skull was even better-adapted to resist stress induced by head-depressors. Considered together with the fact that the center of the arc described by the canines was closer to the jaw-joint in *Smilodon*, our results are consistent with both jaw-closing and neck musculature playing a role in prey dispatch for the placental, as has been previously suggested. However, for *T. atrox*, we conclude that the jaw-adductors probably played no major part in the killing bite. We propose that the metatherian presents a more complete commitment to the already extreme saber-tooth ‘lifestyle’.

## Introduction

Saber-tooth morphology has a deep history, having independently evolved at least twice in the Permo-Triassic among non-mammalian cynodonts and at least five times among Cenozoic mammals, i.e., within the creodont, nimravid, barbourofelid and machairodontine placental, and sparassodont metatherian (a sister group to marsupials) clades [1]–[4]. Consequently, saber-toothed taxa have long figured prominently in analyses and discussions of adaptive convergence.

Of all saber-toothed species, representatives of the felid subfamily Machairodontinae are the best known. There are two widely recognized morphotypes, dirk- and scimitar-tooths. Scimitar-toothed taxa, e.g., *Homotherium serum*, are characterized by shorter, broader upper canines, longer limbs and more gracile physiques. Dirk-tooths, which include the iconic *Smilodon fatalis*, possess longer, more laterally compressed upper canines, and are typically much more robust, with shorter legs and lumbar regions [5]. A third morphotype based on a single machairodontine species has been proposed, incorporating a combination of features [6].

With its extremely long upper canine teeth, powerful neck and forelimb musculature, short limbs, and short lower back, the Miocene-Pliocene metatherian saber-tooth, *Thylacosmilus atrox* appears most similar to specialized dirk-toothed machairodontines such as *S. fatalis*, although separated by at least 125 million years of evolution [7]. It is generally agreed that the bauplan of both *T. atrox* and *S. fatalis* represents an adaptation to the punishing habit of killing relatively large prey and the two are commonly compared in the context of convergent evolution [1], [2], [4], [5], [8]–[16].

Although most authors have long agreed that the dirk-toothed morphotype evolved to preferentially exploit and rapidly kill relatively large prey, our understanding of precisely how they delivered the fatal bite is the subject of one of palaeontology’s longest running debates [4], [12], [17]–[19]. Over the last few decades some consensus has been achieved on the question of killing behaviour, at least with respect to *S. fatalis*. It is now widely recognised that the machairodont’s jaw-closing muscles were relatively small and that at wide gapes mechanical advantage was reduced (i.e., leverage and hence bite reaction or output force was diminished), and, further that the neck muscles likely played an important role in the insertion of the canine teeth, especially in the initial stages of the bite [2], [20], [21]. It is thought that the head-depressing muscles were brought into play first, with the role of the jaw musculature increasing as the gape reduced, possibly with the lower jaw being held against the prey [2], [11]. This *modus operandi*, the ‘canine-shear bite” [11], is essentially an extension of the killing bite applied by living big cats.

Regarding *T. atrox*, however, our understanding of the anatomy of the kill is less clear. Qualitative assessment based on the detailed examination of origin and insertion sites of the primary head-depressors has led to the inference that its neck musculature was relatively even more powerful than that of other saber-tooths, including *S. fatalis* [16]. The same author concluded that the metatherian’s jaws and associated musculature were relatively weaker still. On the other hand, it has also been argued that *T. atrox* may have been capable of a relatively powerful bite [22]. Although it has been suggested that *T. atrox* may have been more specialized than *S. fatalis* on the basis of geometric morphometric studies [23], results from other work based on 2D mandibular force profiling has been interpreted as evidence that the metatherian’s killing behaviour was very similar to that of placental dirk-tooths [22].

In the present study we aim to determine the degree to which killing behaviour may have converged in *Thylacosmilus atrox* and *Smilodon fatalis* through the application of virtual reconstruction techniques and a 3D biomechanical modelling approach known as Finite Element Analysis (FEA). FEA is a powerful non-destructive engineering tool, originally developed for the aerospace industry, but now increasingly used in comparative analyses to predict relative performance in living and fossil taxa, as well as in biomedicine [24]–[36]. Importantly, in addition to facilitating more accurate estimates of reaction forces, such as bite force, with FEA it is also possible to predict, within relative contexts, whether structures are well-adapted to resist hypothesized loads (i.e., simulated behaviors) [35].

This approach has previously been applied in a comparison of biomechanical performance in the crania of *S. fatalis* and a living conical-toothed felid [4], but no FEA-based investigation has included *T. atrox*, or, for that matter, any two saber-toothed species. Our analysis represents a further advance on this earlier work in that we simulate the head-depressing musculature in more detail, estimate maximum gape angles using a new 3D virtual approach, and predict differences in bite force and cranio-mandibular stress at different gapes.

## Materials and Methods

We note that the name *Thylacosmilus atrox* may be a junior synonym of *Achlysictis lelongi* [37] but retain use of the more familiar name until or unless the synonymy becomes more widely accepted. Specimens of *Smilodon fatalis* (FMNH P12418) and *Thylacosmilus atrox* (FMNH P14531, FMNH P14344) were scanned at O’Bleness Memorial Hospital in Athens, OH, USA, using a General Electric LightSpeed Ultra MultiSlice CT scanner with a slice thickness of 625 µm at 120 kV and 200 mA with Extended Hounsfield engaged and bone-reconstruction algorithm. Data were resampled to 300 µm isotropic voxels. For comparative purposes an extant conical-toothed felid, *Panthera pardus* (MM149), was also scanned and modelled. Scanning for this specimen was conducted at the Mater Hospital (Newcastle, Australia) using a Toshiba Aquilion 16 scanner with a slice thickness of 500 µm at 120 kV and 140 mA.

Specimens of both *T. atrox* and *S. fatalis* each retained a single complete upper canine including tooth roots. A complete upper left canine (*T. atrox*) and complete upper right canine (*S. fatalis*), including the tooth roots, were segmented out from the remainder of the crania for both. These were mirrored in Mimics (vers. 13.02) to provide complete upper canines on the opposing sides. For *T. atrox* the cranium was well-preserved in FMNH P14531, but not the dentary, which was taken from P14344. Altogether, for *T. atrox* most of the right upper 3^rd^ premolar, M3 and M4 of FMNH P14531 were missing, and, P14344 comprised a left dentary only. The same mirroring process was applied to reconstruct a complete cranium and mandible. The reconstructed mandible was scaled to fit the slightly larger cranium in Mimics. Upper canines, including tooth roots, were similarly segmented out for *P. pardus*.

**Figure 1:**
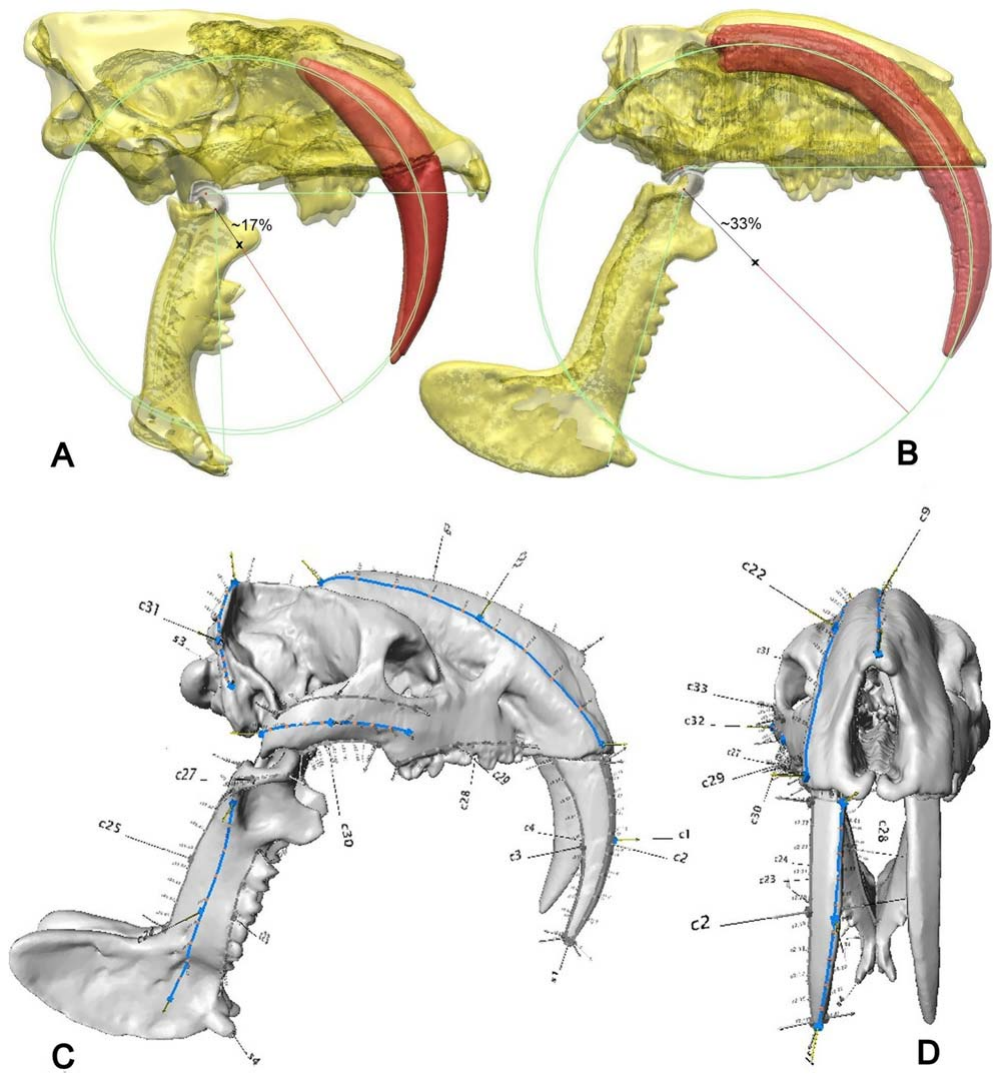
Centres of arcs described by the upper canine teeth. (A) *Smilodon fatalis* and (B) *Thylacosmilus atrox*. The distance of the centre from the jaw joint in *Thylacosmilus atrox* suggests that considerable translation as well as rotation was involved in the insertion of the canine teeth. Landmark positions shown on *Thylacosmilus atrox*. (C) lateral and (D) frontal views of right hand side landmarks. Curves shown in colour relate to Landmark point Von Mises mean stresses. Right hand side landmarks only shown.

**Figure 2:**
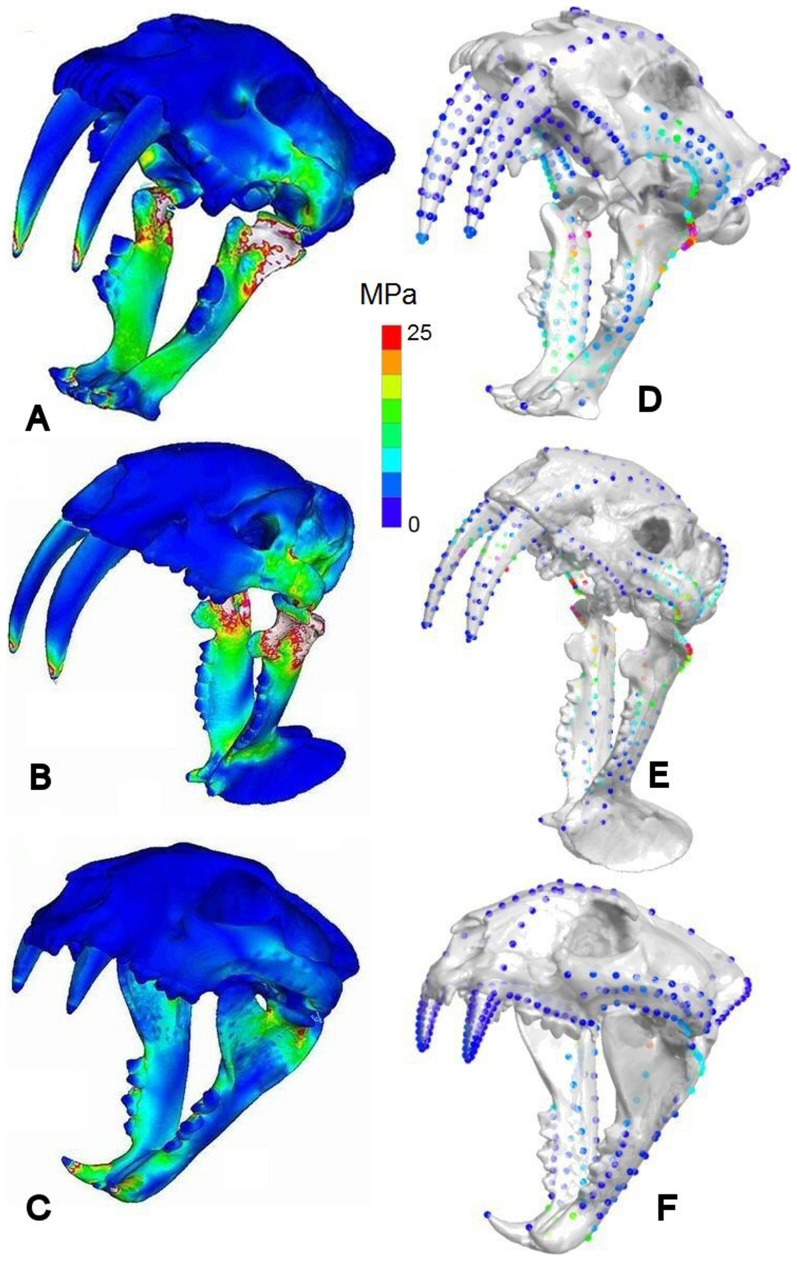
Stress distributions in scaled models for jaw adductor driven bites. Von Mises stress distributions and mean landmark point VM stresses given respectively for (A & D) *Smilodon fatalis*, (B & E) *Thylacosmilus atrox*, (C & F) *Panthera pardus.* MPa = Megapascals. Muscle forces scaled to bite reaction forces predicted on the basis of body mass.

**Figure 3:**
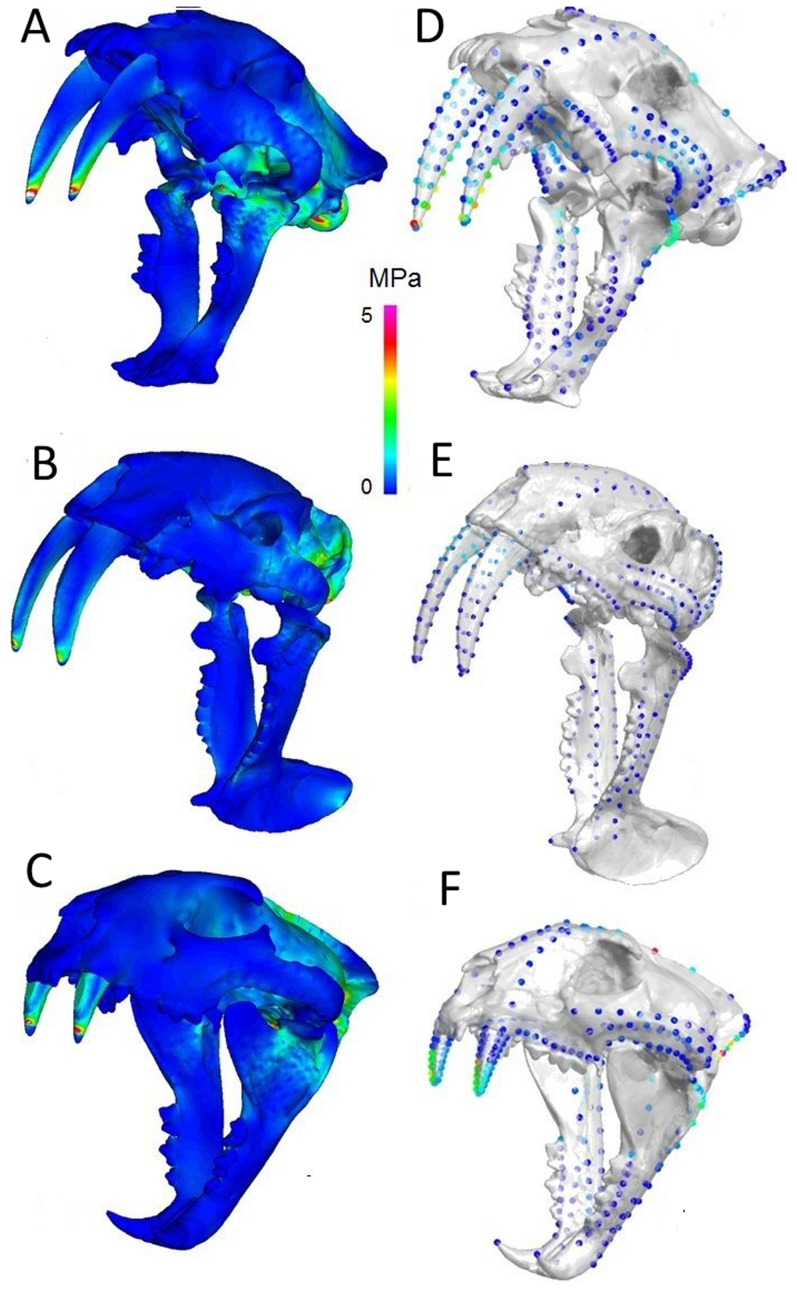
Stress distributions in scaled models for neck muscle driven bites. Von Mises (VM) stress distributions and mean landmark point VM stresses given respectively for (A & D) *Smilodon fatalis*, (B & E) *Thylacosmilus atrox*, (C & F) *Panthera pardus.* MPa = Megapascals. Muscle forces scaled to bite reaction forces predicted on the basis of body mass.

**Figure 4:**
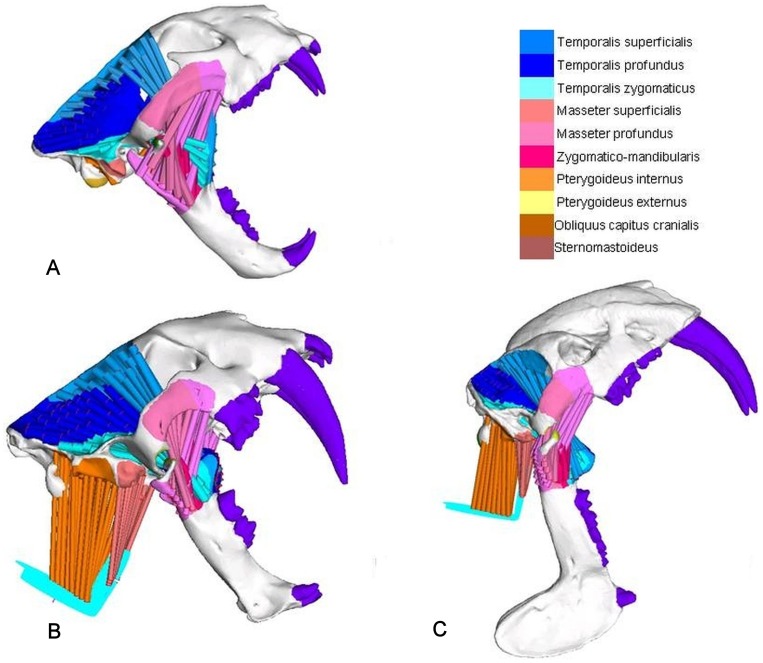
Muscle simulations. (A) jaw-adducting muscles in *Panthera pardus*, (B) head depressing and jaw-adducting muscles in *Smilodon fatalis*, and (C) head-depressing and jaw-adducting muscles in *Thylacosmilus atrox*.

3D Finite Element Models (FEMs) for skulls of *S. fatalis*, *T. atrox*, and the extant conical-toothed felid *Panthera pardus* (leopard) were generated in Mimics with the upper canine teeth and their roots meshed separately so that distinct material properties could be assigned (see Figures 1, 2, 3, 4). External parts of the canines were attached using rigid links [4], [38] and assigned properties for dentine with surface elements assigned properties for enamel [4]. The remainder of the skull was assigned properties for cortical bone [4]. Jaw adducting musculature was assembled using pre-tensioned ‘truss’ elements following previously established protocols [4]. Muscle force estimation also largely followed previous methods (Figure 4) and see below for more detail.

All volumetric meshes comprised four-noded (tet4) ‘brick’ elements and model size was maintained at between 1.63 and 1.65 million ‘bricks’ for each FEM (see Tables S1, S2, S3 in File S1). Volumetric models were imported into Strand7 (vers. 2.4) Finite Element Analysis software. Inputs are given in Tables S1, S2, S3 in File S1. Protocols largely followed those described in recent works [4], [39].

We modelled the head depressing musculature with multiple pre-tensioned trusses and introduced ‘hinges’ to more realistically model muscle action (and see Tables S1, S2, S3 in File S1). These models served as bases for comparative investigations into the influence of gape on bite force and to assess their capacities to sustain loads applied by jaw-closing as opposed to head-depressing muscles.

Muscle forces for the jaw adductors were predicted using the ‘dry-skull’ method for approximating muscle cross-sectional areas [15], [40], [41] following previously applied protocols [4] and see Supporting Information (File S1). This approach does not allow for additional force that might be generated as a consequence of pennation and is thus likely to underestimate actual maximal bite forces [4], [40]. We stress that our primary objective here is to compare relative performance [4], [42]. We do not apply a scaling factor for pennation which would introduce additional assumptions. Muscle origin and insertion areas were approximated on the basis of previous works [16], [43].

For simulations wherein jaw adductors only were recruited constraints were applied at the occipital condyle and the tips of each canine [4], [42]. Previous studies have used a variety of techniques to model the jaw mechanism including constraining a single node against displacement at each temporomandibular joint (TMJ), effectively creating an axis of rotation for the skull [26], [44], [45]. Such techniques have their own implications as the mandible and cranium are not modelled as an articulating structure, and the model therefore does not account for the effect of jaw movement. To overcome the problem of joint articulation, a hinge mechanism was used to simulate jaw operation [4], [36], [39]. Surface plates in the condyle and cotyle region were first selected and tessellated to create a network of fine beams. This was done to minimize stress singularities at the points of attachment of the jaw hinge. The jaw hinge mechanism used rigid links and beams to connect the cranium and mandible, providing a stiff connection between the articulating surfaces and a point on the axis of rotation to which the hinge beam was connected. The beam provided the pivot or hinge in the joint.

The length-tension relationship is a basic property of muscle fibers. Typically, maximum force is generated when fibers are only moderately stretched, and maximizing gape will theoretically compromise bite forces. However, the musculoskeletal configuration of saber-tooths may have allowed them to operate within a more favorable portion of the length–tension curve at larger gapes, as has been demonstrated in some other mammalian taxa [46]. Among living felids there is evidence for increased fiber length in the jaw adductors of species that have wider gapes and that take relatively large prey [47]. This may suggest some mitigation of the tendency to lose muscle force at wide gapes in these species. Our arrangement of truss elements broadly accounts for musculoskeletal features that may have improved performance at wide gapes in saber-tooths [2]. No experimentally derived data is available for the length-tension relationship of the masticatory muscles in large felid or marsupial carnivores and, rather than introduce further assumptions, our modeling effectively accepts that muscle tensions do not decrease with increasing gape. Thus our modeling assumes maximal performance at maximal gapes for jaw closing muscles and we consider it likely that this will result in at least some overestimation of jaw-muscle-driven bite force at wide gapes.

The two major head depressors, M. obliquus capitis and M. sternomastoideus and their origin and insertion points were reconstructed in the FEMs of *S. fatalis*, *T. atrox* and *P. pardus* based on known mastoid anatomy [1], [10], [48]. Crania and mandibles were first rotated about the TMJ to the theoretical maximal gape angle (see below). Rigid links were then used to create an ellipse (major and minor axis in mm: 40 and 26 for *S. fatalis,* 36 and 25 for *T. atrox,* and 30 and 20 for *P. pardus*) and a circle (radius in mm: 30 for *S. fatalis*, 26 for *T. atrox*, and 23 for *P. pardus*) that served as attachment ‘webs’ for the head-flexors. The circular and elliptical webs were kept perpendicular to each other, and their dimensions were proportional to skull length. The centre node in each was fixed in all six degrees of freedom.

Forty truss elements were used to simulate the action of the sternomastoideus muscles and thirty elements were used for the obliquus capitis. In *S. fatalis* each head-depressing truss element was assigned a pretension of 25N. Body mass scaled canine bite force output was then estimated for *T. atrox* and *P. pardus* using *S. fatalis* as reference [4], [15]. Head-flexor muscle recruitment needed to generate this bite force was deduced from the finite element solves for *T. atrox* and *P. pardus*. Jaw adductors were not recruited in these simulations.

Maximum gape angles were predicted on the basis of surface meshes generated from the FEMs. A surface model (in stl format) was exported from the solid model for each specimen. The articulating surfaces of the cranium and mandible were extruded by 1 mm to simulate cartilage covering of the joint. No data is available for articular cartilage thickness in large felids or metatherians and here we use a median value reported for canids [49]. An Iterative Closest Point registration process was then used to fit the cartilage surfaces together [50]. By selecting the regions of the cartilage layers that were in contact when the jaw is opened wide, the rough maximum gape angle can be determined. However, at this point the cartilage layers still overlap considerably. The mandible was then moved until the cartilage layers just touched. A point was placed where cartilage contacted between the mandible and cranium for each TMJ. When connected, these points formed the rotational axis of mandible movement. For each model the mandible was then rotated around this axis until bone-bone contact was achieved at the articulating surfaces of the TMJ. The mandible was then rotated back away from contact with the cranium to account for soft tissue between the angular process on the mandible and the temporal bone to give the final maximum gape angle.

We assessed relative mechanical performance on the basis of visual output of the post-processing software, mean brick stress for selected regions [38], and mean landmark point data [27]. The application of landmarks to reveal mean landmark point VM stresses allows comparisons of values at homologous points in different FEMs [27], [31], [51], thus integrating shape and Finite Element Analyses. We used von Mises (VM) stress because it is a good predictor of material failure in relatively ductile material such as bone [52]. We note, however, that we do not expect to predict actual material failure in these models. Safety factors in mammalian bone can exceed 1000% [53] and, as observed above, our muscle force estimates are likely to be underestimates. Importantly, the approach followed in the present study, like that followed in most similar analyses, is strictly comparative [26], [54]–[57], and it is not actual stress values, but their values and distributions relative to those in other specimens that are of interest [57]. We further observe that we have compared two independently evolved, but at least broadly convergent extremes and have not attempted to compare them in full phylogenetic contexts. This is because there are no living close relatives known for *Thylacosmilus atrox*.

## Results and Discussion

Determining maximal gape is critical to understanding functional adaptation in saber-tooths [32], [58]. Maximal 2D gape angles measured between the upper mesial incisor, jaw-joint, and lower mesial incisor were 87.1° for *S. fatalis*, 105.8° for *T. atrox*, and 72.6° for *P. pardus*. Respectively, these results were 2.5–12.5° less than determined in previous studies for *S. fatalis* [21], [58], slightly higher than the figure of 102° previously suggested for *T. atrox* [9], and, within the 65–70° range predicted for extant felids for *P. pardus* [58].

We found that the center of the arc described by the upper canines (saber-teeth) was considerably closer to the jaw-joint in *S. fatalis* (∼17% of the distance of a line from the fulcrum to the circumference intersecting the center of the arc) as opposed to a value of ∼33% in *T. atrox* for the same measurements. This is more consistent with the canine-shear bite hypothesis in *S. fatalis* than in *T. atrox*, as it means that the canines of the machairodont could have been inserted along a path of less (but not least) resistance, with the mandible rotated about the hinge throughout a killing bite. The far more ventral and anterior position of the center described by the arc of the canines in *T. atrox* means that, for minimal resistance to be maintained, more translation, and hence more input from the cervical musculature, would be necessary while the canines were driven into the prey (Figure 1). However, our findings here suggest that neither species is fully ‘optimized’ in this respect, further supporting the argument that at least some input from the head-depressors was characteristic of the killing bite in both.

Simulations of biting at the canine teeth at maximum gape angles using muscle forces derived on the basis of estimated cross-sectional area [40] gave bite reaction forces of 519 Newtons (N) for *S. fatalis*, 484 N for *P. pardus*, and 38 N for *T. atrox* (Table 1). Estimates of body mass were 259 kg, 68 kg, and 82 kg, respectively. For the fossil taxa these are based on proximal limb data. This was not possible for the leopard as the specimen was represented by the skull only (see Text S1 in File S1).

**Table 1:** Body-mass-adjusted canine bite-force results and mean brick VM stresses for selected regions in a jaw-adductor-driven bite at maximum gape.

	Body mass estimate (kg)	Jaw muscle recruitmentforce (N)	Canine bite force (N)- 2/3rd power rule	Mean VM stress (MPa)		
				Tooth root	Canine-crowns	Rest of the Cranium
*S. fatalis*	259	3785*	519	0.552	2.593	1.074
*T. atrox*	82	24010*	241	0.486	2.311	1.577
*P. pardus*	68	1658*	212	0.397	1.848	0.584

* Muscle forces were back-calculated from FE models that gave the body-mass-scaled bite-force output at canines assuming a 2/3 power relationship. The choice of reference taxon is immaterial in this context and *S. fatalis* was arbitrarily chosen here. kg = kilograms; N = Newtons.

Relative to the conical-toothed cat, jaw-muscle-driven bite forces at wide gapes were relatively weak for *S. fatalis* and extremely weak for *T. atrox*, this despite the fact that our simulations assumed a constant length-tension relationship for muscle fibers at wider gapes. We further note that the estimate for body mass in *T. atrox* used in the present study is conservative and that some authors have predicted figures approaching 120 kg [59], [60], which would make relative jaw-muscle-driven bite force weaker still in the metatherian. *Panthera pardus* was more efficient in converting jaw muscle force to bite reaction force at all gape angles than either saber-tooth. This differential became less marked with decreasing gape, but was still pronounced at smaller angles (see Figure S1 in File S1). At near optimal gape angles of 15° for achieving maximal jaw-muscle-driven bite force, reaction forces at the canines were 1408 N for *S. fatalis*, 1222 N for *P. pardus*, and 585 N for *T. atrox*. The result for this specimen of *S. fatalis* was higher than the 1100 N predicted for a smaller specimen previously modeled using a 3D approach [4], but still very low relative to that predicted for an extant large cat of comparable size, i.e., ∼2906 N for a large male African lion [42].

Although few experimental data are available, and none for large felid or metatherian carnivores, the relationship between vertebrate body mass and bite force is thought to be allometric [15], [61]. All else being equal, the expected relationship between bite reaction force and body mass should be a power function of ^2/3^ because muscle force is proportional to area and body mass is proportional to volume [62]. To account for differences in size between the three species, further simulations were performed with jaw muscle forces scaled to achieve the bite force expected on the basis of size (i.e., assuming a 2/3 power relationship). Under these inputs, our results showed that, relative to *P. pardus*, the crania and mandibles of both saber-tooths would have developed much higher stresses at maximal gapes in jaw-adductor-driven biting, with mean landmark point VM stresses consistently higher for both saber-tooths than for *P. pardus*. This was especially so for *T. atrox*, which recorded values more than four times greater than the leopard in the zygomatic arch. Similarly, mean ‘brick’ element stresses in the cranium were 1.8 times those of *P. pardus* in *S. fatalis* and 2.7 those of *P. pardus* in *T. atrox* (Figure 2, Table 2 and Table S4 in File S1).

**Table 2:** Body-mass-scaled bite-force results and mean brick VM stresses for selected regions in for a cervical-musculature-driven bite.

	Body mass (kg)	Head depressingmuscle force (N)	Canine biteforce (N)	Mean VM stress (MPa)		
				Tooth root	Canine-crowns	Rest of the cranium
*S. fatalis*	259	1750 *	269	0.193	0.585	0.446
*T. atrox*	82	1547*	125	0.19	0.44	0.45
*P. pardus*	68	1076*	110	0.269	0.969	0.574

* Muscle forces were back-calculated from FE models that gave the body-mass-scaled bite-force output at the canines assuming a 2/3 power relationship. *S. fatalis* was arbitrarily used as a reference and head-depressing muscle force used in the model was a hypothetical value. The choice of taxon or value for the reference taxon is immaterial in this context. kg = kilograms; N = Newtons.

Our analysis further shows that in order to achieve bite forces consistent with a 2/3 power relationship at maximum gape, *S. fatalis* would need to recruit 2.3 times the jaw adductor muscle force of *P. pardus*. At maximum gape, *Thylacosmilus atrox* would need to generate 14.5 times the jaw adductor muscle force of *P. pardus* in order to achieve a bite force consistent with its body mass. In contrast, when neck muscle forces only were applied, assuming a 2/3 power relationship between body mass and reaction force at the canines, mean VM landmark point stresses and mean ‘brick’ element stresses were comparable between *S. fatalis* and *P. pardus* and relatively low in *T. atrox* (Figure 3 and Table S5 in File S1).

Although our results are arguably consistent with the canine-shear bite hypothesis for *S. fatalis* [8], the extremely low jaw-adductor-driven bite forces predicted at all gape angles for *T. atrox* suggest that the jaw muscles played an insignificant role in the dispatch of prey by the metatherian. Moreover, our findings suggest that in order to minimize stress on the canine teeth and resistance as the canines were inserted, *T. atrox* needed to move its head considerably further forward and downward relative to the position of the jaw-joint than would *S. fatalis*. This could not be achieved simply through rotation about the jaw-joint and would have required manipulation by cervical and/or other postcranial muscles. The relatively low VM stresses predicted for *T. atrox* in scaled modelling of a neck-muscle-driven bite further support this interpretation.

As has been argued for a range of cranial and postcranial character systems, our simulations provide further evidence for convergence in these two highly derived mammalian predators with respect to the mechanics of the killing bite. At wide gapes, in both species, jaw-muscle-driven bite forces are low, and predicted stress magnitudes and distributions suggest that their crania are less well-adapted to resist high jaw-muscle-driven bite forces, but well-adapted to dissipate loads applied by powerful cervical musculature.

Fewer studies have offered interpretations of hunting and killing behaviour for *T. atrox* than for *S. fatalis*, but a considerable range of possibilities have been forwarded nonetheless. A majority of previous assessments have concluded that *T. atrox* was most likely an ambush predator, however, recent application of geometric morphometric and phylogenetic comparative methods to postcranial data leaves open the possibility that it was capable of sustained; albeit not rapid pursuit [63]. A number of studies have commented on the apparent lack of retractile claws in *T. atrox* and possible limits imposed thereby on its ability to capture and secure prey, leading to speculation that it may have used its head to knock prey over [64]. It has also been argued that the wide gape of *T. atrox* may have allowed it to stab prey at nearly right angles to its body without first needing to restrain it with its claws [9]. Our analyses do not directly assess the likelihood of these suggestions, but we note that the laterally compressed morphology of the canines may have left them vulnerable to breakage in stabbing unsecured prey and that at least some extant ursids are known to capture and immobilize relatively large animals without retractile claws [65]. Among felid, nimravid and barbourofelid saber-tooths there is strong correlation between upper canine length and forelimb robusticity, indicating that powerful forelimbs may be a prerequisite needed to immobilise prey in placental saber-tooths and that this becomes increasingly important as canines become longer and more fragile [66]. Although *T. atrox* was not included in that study there is no doubt that its canines were particularly long and laterally compressed and that its forelimbs were very robust [10], [58].

Analyses based on beam theory have alternatively suggested that *T. atrox* had a weak or powerful jaw-adductor-driven bite [15], [22]. A number of previous authors have suggested that jaw-adductor-driven bite forces may have been particularly weak and that head depressors were especially well-developed, asserting that the head-depressors may have played a particularly important role in driving the canines into prey for the saber-toothed metatherian [9], [10]. Results tendered in the present study provide strong quantitative support for these latter interpretations.

Our findings further suggest that cranio-dental adaptation in *T. atrox* was more specialized than in the machairodontine *S. fatalis*, but the possibility remains open that the metatherian may have converged more completely on other placental saber-tooths not included in the present study. Among these, perhaps the closest in terms of overall cranio-dental and postcranial morphology may have been *Barbourofelis fricki*. Some previous authors have alluded to specific similarities between *T. atrox* and this derived barbourofelid, including possession of a postorbital bar, very long canines, a particularly wide gape, mandibular flanges, and relatively short front and hind limbs [58], [67]. Inclusion of *B. fricki* in future 3D biomechanical analyses could be very informative.

Among placental saber-tooth clades, the evidence now points to independently derived trends toward decreasing jaw-adductor-driven bite force, increasing reliance on head-depressing-musculature, and increasing canine length and forelimb robusticity (i.e., for nimravids, barbourofelids and machairodontines) [2], [4], [66], [68]. A majority of authors have concluded that this suite of features are associated with strong selective pressure for a rapid kill facilitated by precisely directed deep bites into soft tissue that first require effective immobilisation of the prey to limit the risk of damage to the laterally compressed upper canines [4], [66], [68].

Regarding those performance indicators considered in the present study, the very weak jaw-adductor-driven bite forces, cranio-dental anatomy inconsistent with jaw-adductor-driven insertion of the canines along a line of least resistance, and adaptation in the cranium to resist powerful neck-driven-forces present in *T. atrox*, suggest extreme specialization. Whether the metatherian ambushed or ran down its prey, we consider it likely that it was immobilized and secured first because the particularly long and laterally compressed canines would have been especially vulnerable to breakage. This is consistent with evidence for powerful and flexible forelimb musculature, together with other postcranial adaptations for stability [10].

A final point to be considered here is that the flattened canines of *T. atrox* may have required less force to insert than did those of *S. fatalis*. On this basis it could reasonably be argued that the jaw adductors may still have played a significant role in the kill. We would maintain, however, that both our biomechanical evidence suggesting that the cranium was much better adapted to resist forces incurred by a neck-driven-bite, together with that showing that canine morphology was not ‘optimized’ for a jaw-adductor-driven bite, remains inconsistent with a significant role for the jaw adductors in the kill.

The fossil record evidencing distinct structural intermediates between more generalized sparassodonts and *T. atrox* remains poor, despite the recent discovery of new material [69]. However, regardless of the process through which the distinctive morphology of *T. atrox* was derived, we suggest that in the craniodental mechanics of the killing bite that define the dirk-toothed morphotype, the metatherian represents a further extreme in functional adaptation relative to that of *S. fatalis*. Our results further support the contention that despite far lower species richness and greater geographic restriction over time relative to their placental counterparts, metatherian carnivores achieved broadly comparable diversity in terms of behaviour and craniodental morphology [14], [23].

## Supporting Information

File S1 **Text S1,** Body mass estimates. **Figure S1,** Variations in canine bite reaction force (BF) and jaw muscle recruitment (MR) with changing gape angle. **Table S1,** Inputs for Finite Element Model of Smilodon fatalis. **Table S2,** Inputs for Finite Element Model of *Thylacosmilus atrox.* **Table S3,** Inputs for Finite Element Model of *Panthera pardus.* **Table S4,** Mean landmark point Von Mises stresses for jaw-muscle-driven bite scaled to body mass. **Table S5,** Mean landmark point Von Mises stresses for neck-muscle-driven bite scaled to body mass.
(DOCX)
