# Supplementary material for: Comparative Biomechanical Modeling of Metatherian and Placental Saber-Tooths: A Different Kind of Bite for an Extreme Pouched Predator
Source: PLoS One. 2013 Jun 26;8(6):e66888. doi: 10.1371/journal.pone.0066888 (PMC3694156; doi:10.1371/journal.pone.0066888)
Supplement: File S1 — Text S1, Body mass estimates. Figure S1, Variations in canine bite reaction force (BF) and jaw muscle recruitment (MR) with changing gape angle. Table S1, Inputs for Finite Element Model of Smilodon fatalis. Table S2, Inputs for Finite Element Model of Thylacosmilus atrox. Table S3, Inputs for Finite Element Model of Panthera pardus. Table S4, Mean landmark point Von Mises stresses for jaw-muscle-driven bite scaled to body mass. Table S5, Mean landmark point Von Mises stresses for neck-muscle-driven bite scaled to body mass. (DOCX) [file pone.0066888.s001.docx]

**SUPPORTING INFORMATION (SI)**

**“Comparative biomechanical modeling of metatherian and placental saber-tooths: A different kind of bite for an extreme pouched predator”**

**Text S1**

**Body mass**

The postcranial skeleton of *Smilodon fatalis* is extremely robust and far more massive than that of any extant cat [[1](#_ENREF_1)]. Recent body mass estimates based on postcranial data suggest that it was up to around 280 kg, comparable to the very largest living felid subspecies, the Siberian tiger [[2](#_ENREF_2)]. Thus, estimates based on cranial dimensions, deduced on the basis of regression data from living felids, almost certainly underestimate its body weight. The body mass estimate for the specimen of *S. fatalis* included in this study was generated using a previously applied approach [[3](#_ENREF_3)], i.e., geometric similitude was assumed between the specimen used in our study (FMNH P 12418) and the only specimen of *S. fatalis* for which a near complete skeleton is known (LACM PMS 1-1). That is, body mass for this near-complete specimen was calculated on the basis of proximal limb bone minimum circumference data and a 2/3rd power relationship was then assumed between the basal skull length and the body mass. The body mass estimate for FMNH P 12418 using this approach was ~ 259 kg. The body mass estimate of ~ 82 for *T. atrox* was obtained directly from the literature [[4](#_ENREF_4)]. This figure was a mean derived on the basis of three different quantitative approaches for this specimen [[5](#_ENREF_5)]. For *P. pardus* the body mass estimate of ~68 kg was obtained on the basis of skull-length/body-mass regression data from extant felids [[6](#_ENREF_6)].

**Figure S1.** Variations in canine bite reaction force (BF) and jaw muscle recruitment (MR) with changing gape angle in *Smilodon fatalis*, *Thylacosmilus atrox* and *Panthera pardus*. N = Newtons.

| **Table S1**  Inputs for Finite Element Model (FEM) of *Smilodon fatalis* (FMNH P12418) | | | | | |
| --- | --- | --- | --- | --- | --- |
|  |  |  |  |  |  |
| Force /Muscle area (KPa) [[7](#_ENREF_7)] | 300 | Basal-condylar length (mm) | | 310.74 |  |
| Unilateral X-sectional area - Temporalis (mm^2) | 4481.28 | Cranial width at zygomatic arch (mm) | | 196.91 |  |
| Unilateral X-sectional area - Masseter (mm^2) | 3796.17 | Mandible length (mm) | | 203.98 |  |
|  |  | Mandible width at condyles (mm) | | 163.28 |  |
| Unilateral Temporalis muscle force (N) | 1344.38 | Total skull bone volume (mm^3) | | 1.55E+06 |  |
| Unilateral Masseteric muscle force(N) | 1138.85 | Surface area (mm^2) | | 3.26E+05 |  |
| Total muscle force (N) | 2483.24 | Number of tet4 brick elements in FE model | | 1643322 |  |
|  |  |  |  |  |  |
| Jaw muscle X-sectional areas (mm^2) | | % area occupied | Truss elements on each side of the skull | Force/beam (N) | Truss diameter (mm) |
| Temporalis superficialis | 8175.48 | 16.78 | 17 | 19.77 | 9.16 |
| Temporalis profundus | 18119.12 | 37.18 | 37 | 19.77 | 9.16 |
| Temporalis zygomaticus | 6950.105 | 14.26 | 14 | 19.77 | 9.16 |
| Masseter superficialis | 4988.14 | 10.24 | 10 | 39.27 | 6.46 |
| Masseter profundus | 5030.212 | 10.32 | 11 | 39.27 | 6.46 |
| Zygomatico-mandibularis | 3890.397 | 7.98 | 8 | 39.27 | 6.46 |
| Pterygoideus internus | 1061.358 | 2.18 | 2 | balancing beam | 7.00 |
| Pterygoideus externus | 515.6937 | 1.06 | 1 | balancing beam | 7.00 |
| Head-depressing muscle X-sectional areas (mm^2) | | Truss elements on either side of the skull | Force/beam (N) | Truss diameter (mm) |  |
| Sternomastoideus | 1926.791 | 40 | 25 | 5 |  |
| Obliquus capitis | 6688.694 | 30 | 25 | 5 |  |
| ‘Brick’ material properties [[8](#_ENREF_8),[9](#_ENREF_9)] | Young's modulus (GPa) | Density (T/mm^3) |  |  |  |
| Cranium and Mandible | 21.734 | 1.86E-09 |  |  |  |
| Dentine | 32.704 | 2.526E-09 |  |  |  |
| Enamel | 38.575 | 2.861E-09 |  |  |  |
|  |  |  |  |  |  |
| Beam material properties | Young's modulus (MPa) | Density (T/mm^3) | Diameter (mm) |  |  |
| Muscle trusses | 1.00E-01 | 1.01E-09 | table above |  |  |
| Occipital beams | Structural steel (Strand7 material library SS4100-1998) | | 5.00 |  |  |
| Cotyle beams |  |  | 0.50 |  |  |
| Condyle beams |  |  | 0.50 |  |  |
| Hinge beam |  |  | 5.00 |  |  |
| Origin and Insertion beams |  |  | 0.50 |  |  |
|  | | | |  |  |

**Table S2**

| Inputs for Finite Element Model (FEM) of *Thylacosmilus atrox* (FMNH P14531 and FMNH P14344) | | | | | |
| --- | --- | --- | --- | --- | --- |
|  |  |  |  |  |  |
| Force /Muscle area (KPa) [[7](#_ENREF_7)] | 300 | Basal-condylar length (mm) | | 219.141 |  |
| Unilateral X-sectional area - Temporalis (mm^2) | 1717.47 | Cranial width at Zygomatic arch, mm | | 139.305 |  |
| Unilateral X-sectional area - Masseter (mm^2) | 1748.08 | Mandible length (anterior dentary to condyle), mm | | 192.851 |  |
|  |  | Mandible width at condyles, mm | | 132.842 |  |
| Unilateral Temporalis muscle force (N) | 515.24 | Total skull bone volume (mm^3) | | 1.14E+06 |  |
| Unilateral Masseteric muscle force(N) | 524.42 | Surface area (mm^2) | | 2.66E+05 |  |
| Total muscle force (N) | 1039.67 | Number of tet4 elements in FE model | | 1643322 |  |
|  |  |  |  |  |  |
| Jaw muscle cross-sectional areas (mm^2) | | % area occupied | Truss elements on either side of the skull | Force/beam (N) | Truss diameter (mm) |
| Temporalis superficialis | 4013.01 | 12.96 | 13 | 9.37 | 6.31 |
| Temporalis profundus | 9091.46 | 29.36 | 29 | 9.37 | 6.31 |
| Temporalis zygomaticus | 4055.56 | 13.10 | 13 | 9.37 | 6.31 |
| Masseter superficialis | 5424.58 | 17.52 | 17 | 12.79 | 7.37 |
| Masseter profundus | 4527.18 | 14.62 | 15 | 12.79 | 7.37 |
| Zygomatico-mandibularis | 2722.71 | 8.79 | 9 | 12.79 | 7.37 |
| Pterygoideus internus | 784.875 | 2.53 | 3 | balancing beam | 7.00 |
| Pterygoideus externus | 343.757 | 1.11 | 1 | balancing beam | 7.00 |
| Head-depressing cross-sectional areas (mm^2) | | Truss elements on either side of the skull | Force/beam (N) | Truss diameter (mm) |  |
| Sternomastoideus | 786.45 | 40 | 25 | 5 |  |
| Obliquus capitis | 2897.2 | 30 | 25 | 5 |  |
| Brick material properties [[8](#_ENREF_8),[9](#_ENREF_9)] | Young's modulus (GPa) | Density (T/mm^3) |  |  |  |
| Cranium and mandible | 21.734 | 1.86E-09 |  |  |  |
| Dentine | 32.704 | 2.526E-09 |  |  |  |
| Enamel | 38.575 | 2.861E-09 |  |  |  |
|  |  |  |  |  |  |
| Beam material properties | Young's modulus (MPa) | Density (T/mm^3) | Diameter (mm) |  |  |
| Muscle trusses | 1.00E-01 | 1.01E-09 | table above |  |  |
| Occipital beams | Structural steel (Strand7 material library SS4100-1998) | | 5.00 |  |  |
| Cotyle beams |  |  | 0.50 |  |  |
| Condyle beams |  |  | 0.50 |  |  |
| Hinge beam |  |  | 5.00 |  |  |
| Origin and Insertion beams |  |  | 0.50 |  |  |
|  | | | | | |

**Table S3**

| Inputs for Finite Element Model (FEM) of *Panthera pardus* (MM149) | | | | | |
| --- | --- | --- | --- | --- | --- |
|  |  |  |  |  |  |
| Force /Muscle area (KPa) [[7](#_ENREF_7)] | 300 | Basal-condylar length (mm) | | 216.974 |  |
| Unilateral X-sectional area - Temporalis (mm^2) | 3002.29 | Cranial width at Zygomatic arch, mm | | 155.839 |  |
| Unilateral X-sectional area - Masseter (mm^2) | 1580.24 | Mandible length (anterior dentary to condyle), mm | | 159.145 |  |
|  |  | Mandible width at condyles, mm | | 137.753 |  |
| Unilateral Temporalis muscle force (N) | 900.69 | Total skull bone volume (mm^3) | | 5.43E+05 |  |
| Unilateral Masseteric muscle force (N) | 474.07 | Surface area (mm^2) | | 1.65E+05 |  |
| Total muscle force (N) | 1374.76 | Number of tet4 elements in FE model | | 1631130 |  |
| Jaw muscle cross-sectional areas (mm^2) | | % area occupied | Truss elements on either side of the skull | Force/beam (N) | Truss diameter (mm) |
| Temporalis superficialis | 12296.8 | 32.60702308 | 33 | 14.39 | 7.82 |
| Temporalis profundus | 6117.92 | 16.22268872 | 16 | 14.39 | 7.82 |
| Temporalis zygomaticus | 4920.82 | 13.04837774 | 13 | 14.39 | 7.82 |
| Masseter superficialis | 5589.89 | 14.82252881 | 15 | 23.53 | 9.99 |
| Masseter profundus | 4172.99 | 11.06538134 | 11 | 23.53 | 9.99 |
| Zygomatico-mandibularis | 3065.64 | 8.129057499 | 8 | 23.53 | 9.99 |
| Pterygoideus internus | 1087.61 | 2.883979928 | 3 | balancing beam | 7.00 |
| Pterygoideus externus | 460.451 | 1.220962884 | 1 | balancing beam | 7.00 |
| Head-depressing muscle cross-sectional areas (mm^2) | | Truss elements on either side of the skull | Force/beam (N) | Truss diameter (mm) |  |
| Sternomastoideus | 1273.25 | 40 | 25 | 5 |  |
| Obliquus capitis | 2672.62 | 30 | 25 | 5 |  |
| Brick material properties[[8](#_ENREF_8),[9](#_ENREF_9)] | Young's modulus (GPa) | Density (T/mm^3) |  |  |  |
| Cranium and mandible | 21.734 | 1.86E-09 |  |  |  |
| Dentine | 32.704 | 2.526E-09 |  |  |  |
| Enamel | 38.575 | 2.861E-09 |  |  |  |

|  |  |  |
| --- | --- | --- |
| Beam material properties | Young's modulus (MPa) | Density (T/mm^3) |
| Muscle trusses | 1.00E-01 | 1.01E-09 |
| Occipital beams | Structural steel (Strand7 material library SS4100-1998) | |
| Cotyle beams |  |  |
| Condyle beams |  |  |
| Hinge beam |  |  |
| Origin and Insertion beams |  |  |

**Table S4**

Mean landmark point Von Mises (VM) stresses for jaw-muscle-driven bite scaled to body mass. 345 homologous landmarks were placed on the surfaces of the three models using Landmark.exe following recently published methods [[10](#_ENREF_10)] and see Figure 1 of article. Mean landmark point Von Mises stresses were calculated by taking the mean VM stress of the three closest ‘brick’ elements to each of these landmarks [[10](#_ENREF_10)]. This allows direct comparison of results of FEAs of the three FEMs under different biting regimes. Ant Edge C = anterior edge of canine, Distal C = distal edge of canine, Lat Zygo Arch = lateral zygomatic arch, Lat Mand = lateral mandible. **M** = mean, **SD** = standard deviation, **Mx** = maximum.

| \| **Species** \| **Ant Edge C** \| **Sagittal Line** \| **Distal C** \| **Lat Zygo Arch** \| **Nuchal Crest** \| **Lat Mand** \| \| --- \| --- \| --- \| --- \| --- \| --- \| --- \| \| *P. pardus* ***M*** \| 1.063 \| 0.157 \| 0.186 \| 1.803 \| 0.254 \| 4.527 \| \| *P. pardus* ***SD*** \| 0.896 \| 0.126 \| 0.067 \| 0.986 \| 0.207 \| 5.933 \| \| *P.pardus* ***Mx*** \| 2.375 \| 0.472 \| 0.288 \| 3.413 \| 0.626 \| 16.808 \| \| *S. fatalis* ***M*** \| 1.535 \| 0.347 \| 0.475 \| 4.307 \| 0.286 \| 12.356 \| \| *S. fatalis* ***SD*** \| 1.141 \| 0.157 \| 0.187 \| 1.822 \| 0.373 \| 7.727 \| \| *S. fatalis* ***Mx*** \| 4.212 \| 0.584 \| 0.736 \| 7.744 \| 1.125 \| 31.884 \| \| *T. atrox* ***M*** \| 2.114 \| 0.239 \| 1.167 \| 7.908 \| 4.182 \| 7.473 \| \| *T. atrox* ***SD*** \| 2.042 \| 0.124 \| 1.217 \| 3.818 \| 2.705 \| 8.147 \| \| *T. atrox* ***Mx*** \| 7.502 \| 0.410 \| 4.133 \| 13.634 \| 9.020 \| 21.125 \| |  |  |  |  |  |  |
| --- | --- | --- | --- | --- | --- | --- | --- | --- | --- | --- | --- | --- | --- | --- | --- | --- | --- | --- | --- | --- | --- | --- | --- | --- | --- | --- | --- | --- | --- | --- | --- | --- | --- | --- | --- | --- | --- | --- | --- | --- | --- | --- | --- | --- | --- | --- | --- | --- | --- | --- | --- | --- | --- | --- | --- | --- | --- | --- | --- | --- | --- | --- | --- | --- | --- | --- | --- | --- | --- | --- | --- | --- | --- | --- | --- | --- |
|  |  |  |  |  |  |  |
|  |  |  |  |  |  |  |
| **Table S5**  Mean landmark point Von Mises stresses for neck-muscle-driven bite scaled to body mass. Ant Edge C = anterior edge of canine, Distal C = distal edge of canine, Lat Zygo Arch = lateral zygomatic arch, Lat Mand = lateral mandible. **M** = mean, **SD** = standard deviation, **Mx** = maximum.   \| **Species** \| **Ant Edge C** \| **Sagittal Line** \| **Distal C** \| **Lat Zygo Arch** \| **Nuchal Crest** \| **Lat Mand** \| \| --- \| --- \| --- \| --- \| --- \| --- \| --- \| \| *P. pardus M* \| 1.523 \| 0.998 \| 0.516 \| 0.251 \| 2.685 \| 0.204 \| \| *P. pardus SD* \| 1.218 \| 1.609 \| 0.292 \| 0.146 \| 2.475 \| 0.234 \| \| *P. pardus Mx* \| 3.325 \| 5.365 \| 1.156 \| 0.488 \| 8.392 \| 0.731 \| \| *S. fatalis M* \| 1.803 \| 0.530 \| 0.628 \| 0.455 \| 0.566 \| 0.412 \| \| *S. fatalis SD* \| 3.673 \| 0.580 \| 0.415 \| 0.228 \| 0.456 \| 0.804 \| \| *S. fatalis Mx* \| 12.208 \| 1.701 \| 1.414 \| 1.024 \| 1.387 \| 2.600 \| \| *T. atrox M* \| 0.325 \| 0.110 \| 0.197 \| 0.265 \| 0.432 \| 0.189 \| \| *T. atrox SD* \| 0.321 \| 0.128 \| 0.265 \| 0.186 \| 0.244 \| 0.087 \| \| *T. atrox Mx* \| 0.743 \| 0.451 \| 0.921 \| 0.590 \| 0.827 \| 0.289 \| |  |  |  |  |  |  |

1. Wroe S, Lowry MB, Anton M (2008) How to build a mammalian super-predator. Zoology 111: 196-203.

2. Christiansen P, Harris JM (2005) Body size of *Smilodon* (Mammalia: Felidae). Journal of Morphology 266: 369-384.

3. McHenry CR, Wroe S, Clausen PD, Moreno K, Cunningham E (2007) Supermodeled sabercat, predatory behavior in *Smilodon fatalis* revealed by high-resolution 3D computer simulation. Proceedings of the National Academy of Sciences (USA) 104: 16010-16015.

4. Argot C (2004) Functional-adaptive features and palaeobiologic implications of the postcranial skeleton of the late Miocene sabretooth borhyaenoid *Thylacosmilus atrox* (Metatheria). Alcheringa 28: 229-266.

5. Anyonge, W. (1993) Body mass in large extant and extinct carnivores. Journal of Zoology (London) 231: 339-350.

6. Van Valkenburgh B (1990) Skeletal and dental predictors of body mass in carnivores. In: Damuth J, MacFadden BJ, editors. Body size in mammalian paleobiology: Estimation and biological applications. Cambridge: Cambridge University Press. pp. 181-205.

7. Weijs WA, Hillen B (1985) Cross-sectional area and estimated intrinsic strength of the human jaw muscles. Acta Morphol Neerl Scand 23: 267-274.

8. Rho JY, Hobatho MC, Ashman RB (1995) Relations of mechanical properties to density and CT numbers in human bone. Medical Engineering and Physiology 17: 347-355.

9. Alexander RM (1980) Forces in animal joints. Engineering Medical 9: 93-97.

10. Parr W, Wroe S, Chamoli U, Richards HS, McCurry M, et al. (2012) Toward integration of geometric morphometrics and computational biomechanics: New methods for 3D virtual reconstruction and quantitative analysis of Finite Element Models. Journal of Theoretical Biology 301: 1-14.
